# Detrimental effect of antiepileptic drugs dose in pediatric children with epilepsy in Saudi Arabia: A prospective cohort study

**DOI:** 10.1097/MD.0000000000026478

**Published:** 2021-07-02

**Authors:** Badriyah S. Alotaibi, Abdulaziz A. Alodhayani, Ashraf Alwan, Khalid Nijr Alotaibi, Brahim Tabarki Melaiki, Aljawharah Mohammad Almadhi, Lulwah Haitham Alfares, Nahlah Ahmed Alalkami

**Affiliations:** a College of Pharmacy, Princess Nourah Bint Abdulrahman University, Riyadh, Saudi Arabia; b Department of Family and Community Medicine, College of Medicine, King Saud University, Riyadh, Saudi Arabia; c Department of Pharmaceutical Services, Prince Sultan Military Medical City, Riyadh, Saudi Arabia; d Pediatric Neurology, Prince Sultan Military Medical City, Department of Pharmaceutical Services, Riyadh, Saudi Arabia.

**Keywords:** adverse effects, antiepileptic drugs, dose alterations, medication

## Abstract

This study aims to evaluate the effect of dose titration for different oral antiepileptic medications among children with epilepsy in Riyadh, Saudi Arabia.

A single-center prospective pilot, cohort study was undertaken at a tertiary hospital in Riyadh, Saudi Arabia. All medical records of pediatric patients below the age of 14 years of age who has been newly diagnosed with epilepsy by attending a medical specialist or on a new epileptic treatment plans were enrolled in the study.

A total of 76 epileptic patients were screened for 3 months’ period and 48 patients were included in this study. Out of the 48 patients, 31 patients followed the regular practice in the titration processes and 17 patients were in the British national formulary (BNF) guideline. Fifteen children who were on monotherapy of levetiracetam were in regular practice guideline experienced poor seizure control with a recorded number of seizure incidence (n = 10). The patient in regular practice guidelines using a combination therapy of phenytoin and levetiracetam were experiencing some behavioral disturbance and sedation effect. Seventeen patients followed in the BNF guideline who were on levetiracetam were experienced less adverse effect (n = 2) with no behavioral changes.

The group who followed the regular practice found having a greater incidence of documented adverse effects compared to the patients following the BNF guideline. The titrating antiepileptic medication has a detrimental effect on the pediatric population as observed in this study.

## 1 Introduction

Epilepsy is the recurrence of at least two reflex seizures that involve an alteration in patient's level of motor and sensory consciousness and are not due to any identifiable cause.^[1]^ A variety of epidemiological studies of epilepsy have been restricted to address the epilepsy incidence and prevalence in children before 16 years of age due to a different age range of subjects, epilepsy definitions, and methods of case ascertainment.^[2,3]^ Epilepsy incidence is the number of novel cases of epilepsy per year in a defined population and is usually depicted as cases per 100,000.^[3]^ The incidence is reliably stated to be at its peak in the initial year of life and drops to adult levels during the last stage of the first decade.^[3]^ Globally, the epilepsy incidence rate in infancy (1–12 months of age) is 144 per 100,000 person-years and 58 per 100,000 for 1 to 10 years.^[4]^ In Saudi Arabia, studies on epilepsy are limited, and most of the evidence was given by hospitals,^[5]^ which may perhaps not be representative of the real prevalence of the disease. In one community- based study, the prevalence of epilepsy in Saudi Arabia was observed to be similar when compared with Western countries (6.24 per 1000 people).^[6]^

The incidence of epilepsy syndromes and prevalence of particular seizure types are well documented. Epidemiological based studies conducted a minor, but reliable, on the prevalence of focal seizures parallel to the generalized seizures.^[7,8]^ Around 1/3^rd^ of children having epilepsy may be assigned to a particular epilepsy syndrome.^[3]^ Since the introduction of phenobarbital early in the 21^st^ century, great progress has been made in the area of antiepileptic drugs (AEDs) development.^[9]^ Currently, two generations of AEDs are in use. The old generation is represented by drugs like; Valproate (VPA), Carbamazepine (CBZ), Phenytoin (PHT), and Phenobarbital (PHB). While the new generation is represented by: Lamotrigine (LMT), Levetiracetam (LVT), Oxcarbazepine (OXC), Topiramate (TPM), Clonazepam (CLNZ), Vigabatrin (VGB), Pregabalin (PGB), Rufinamide (RFM), and Lacosamide (LAC).^[10]^

The British National Formulary (BNF) recommended guidelines to minimize adverse effects of AEDs and reduction of seizures through unbiased individualization of the patients and exact drug prescription.^[11]^ The BNF guideline provides recommendations about significant adverse effects, withdrawal recommendations, precautions and any requirement for blood testing.^[11]^ It has been recommended that AEDs may exhibit unfavorable side effects. These side effects commonly due to concentration-related drug therapy for example: ataxia, dizziness, stomach upset or blurred vision which are mostly brief and mild, and often they can be minimized by adjusting the dose or direction of use.^[12]^ The incidence of side effect is extremely higher in the pediatric population because of several changes in pharmacokinetic parameters and pharmacodynamic responses occur with maturation throughout childhood.^[13,14]^ Similar effects can be induced by slower rates of liver metabolism in neonate and higher activity of hepatic microsomal enzymes in children especially between 2–4 years of age due to relatively larger liver size in comparison to total body weight which requires higher maintenance doses compared to adults.^[15]^ These adverse effects were found to be more pronounced among patients on old AEDs compared to their counterparts on new AEDs.^[15,16]^

Dose Titration is a continual dose adjustment that depends upon the patient response to achieve optimal clinical control of the disease with minimal adverse events. The purpose of dose titration is to find the perfect balance of a particular medicine for the patient. To achieve this, a drug dose titration needs to be individualized. Dose titration is commonly used in most AEDs to decrease the adverse events and improve tolerability.^[17]^ Measuring antiepileptic drug concentration level (therapeutic drug monitoring) is frequently used to optimize individual drug therapy. Monotherapy titration is titrating a single drug slowly and gradually to the desired dose.^[18]^ For example, in medications such as: CBZ, LMT, TPM, gradual dose titration was preferred because of their high side effect. Meanwhile, PHT, OXC, VPA and LVT are recommended medications that can be started at an effective dose in case of multiple recent seizures or having closed seizure attacks.^[19]^ Monotherapy medication regimen usually successfully manages half of the epilepsy patients, but if the patient fails to become seizure free with the initial AED or a novel adjunctive AED's therapy is suggested.^[19,20]^ Polytherapy titration is using more than one medication by holding the current medication at a constant dose and then gradually titrate the new adjunctive medication to the targeted dose.^[19]^

Most pediatric patients with predominant seizure type commonly use AEDs unlicensed, due to the underrepresentation of children in well-designed, properly conducted Randomized Controlled Trials (RCTs) that address the efficacy and tolerability of AEDs which makes optimization of pharmacotherapy among this population challenging. As a result, lack of guidance regarding initiation and dose titration of AED monotherapies or in adding new adjunctive AED's into a regimen is a major concern in pediatric population. Lack of rigorous opposing effects of data make it difficult to progress an evidence-based guideline intended at recognizing the overall optimal recommended initial-monotherapy of AED.

There is an alarming lack of well-designed, properly conducted RCTs for children and the majority of relevant studies have significant methodological problems that limit their applicability. Multicenter efforts are required to design, conduct and examine future clinical relevant RCTs that can answer several questions. The ultimate choice of an AED for any individual patient with newly diagnosed or untreated epilepsy has to comprise consideration of the efficacy and effectiveness for each AED along with other variables like the safety and tolerability profile, pharmacokinetic properties, formulations.

This study performed to evaluate the effect of dose titration for different oral antiepileptic medications in the PSMMC formulary (CBZ, OXC, CLNZ, PHT, PHB, VPA, LMT, LVT, TPM, VGB, PGB, RFM, and LAC) in enhancing and controlling the patient's disease state and tolerability. The main targeted outcome is the effectiveness of dose titration by measuring seizure control. The subsidiary targeted outcome is to improve the safety of antiepileptic drugs by lessening the side effect with gradual dose titration.

## 2 Methodology

### 2.1 Study design and setting

A single-center prospective pilot, cohort study was undertaken at Prince sultan military medical city (PSMMC) Riyadh, Saudi Arabia. The type of antiepileptic drugs was classified into conventional AEDs (1^st^ generation) and newer AEDs (2^nd^ generation). The conventional AEDs which was prescribed in the PSMMC include: CBZ, VPA, PHT, and PHB. While the newer AEDs prescribed are: LMT, LVT OXC, TPM, CLNZ, VGB, PGB, RFM, and LAC. Types of seizures were classified based on the International League Against Epilepsy classification into three main types: focal (partial) seizures, generalized seizures and undetermined whether focal or generalized seizures.

### 2.2 Participants

The population comprised of all children with epilepsy who were treated at the neurology outpatient clinics in PSMMC, Riyadh, Saudi Arabia. All patients were monitored for the safety and efficacy of their antiepileptic drugs each visit. A medical record of pediatric patients below 14 years’ old who have been newly diagnosed with epilepsy or on a new epileptic treatment plan (such as: increasing the dose of the current antiepileptic drug or adding a novel antiepileptic drug to the treatment regimen) were enrolled in the study regardless of type, gender, and ethnicity. And patient above 14 years and patients that use antiepileptic drugs for other reasons than to control seizures were excluded from the study. The study conducted for about 6 months in PSMMC which is a tertiary care center. The epileptic cases are diagnosed in the hospital by medical specialists and then referred to pediatric neurology department. This study was permitted by the PSMMC Ethics Service on 16/10/2018. The parents were subsequently requested to indicate their approval for their children to enter this study by signing a dated consent form.

### 2.3 Variables and measures

The research team designed a case report form (CRF) for the purposes of this study (Appendix B). The medical notes were examined to establish:

1. Date of entry into the study
2. Age at study entry, in years and months
3. Weight and height
4. Seizure type
5. Epilepsy syndrome (if identified); epilepsy category
6. Current anticonvulsant(s) with doses and formulation
7. Date of commencement of current anticonvulsant drugs

Subsequent follow-up such as current anticonvulsant(s) with the doses and formulation, any changes to the treatment plan (either increasing the dose of current antiepileptic drug or adding a new antiepileptic drug to the treatment regimen) were filed.

### 2.4 Statistical analysis

Data were entered and analyzed using SPSS windows v. 23 (Chicago, Illinois). All continuous data were presented as mean ± SD while categorical data were presented as frequencies and percentages. The data analyses focused on the presentation of descriptive statistics.

## 3 Results

**Figure 1:**
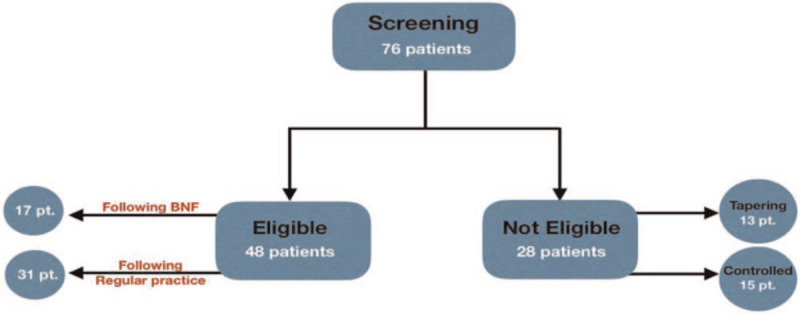
. Screening process of epileptic patients.

A total of 48 patients were recruited into this study. Out of the 48 patients, 31 (64.5%) patients followed the regular practice in the titration process which did not follow any specific guideline, and 17 (34.5%) patients were in the BNF guideline recommendation process (Fig. 1).

The demographic characteristics of the patients were shown in the Table 1. A total of 75 AEDs was recorded in 48 patients, consisting of those on levetiracetam (n = 28, 37.3%), lamotrigine (n = 2, 26%), valproate (n = 9, 12%), topiramate (n = 14, 18.7%), carbamazepine (n = 8, 10.7%), oxcarbazepine (n = 4, 5.3%), phenytoin (n = 22.6%), phenobarbital (n = 6, 8%), and rufinamide (n = 2, 2.6%). Patient characteristics were generally consistent across index AED groups (Table 2); exceptions with a lower number of included patients that are younger than 12 months, the proportion of males was slightly higher than the female by 1.3:1. Thirty-five AEDs were recorded among patients who followed the regular practice and 40 AEDs from the patients who followed the BNF guidelines.

**Table 1:** Age, gender details of patients following regular antiepileptic drug therapy and BNF guidelines.

Patients following				
	Regular practice		BNF guidelines	
Age	Male	Female	Male	Female
> 5 yrs	8 (44.4)	1 (11.1)	4 (44.4)	2 (40)
6–10 yrs	7 (38.8)	5 (55.5)	3 (33.3)	2 (40)
11–14 yrs	3 (16.6)	3 (33.3)	2 (22.2)	1 (20)
Total (N = 41)	18 (43.9)	9 (21.9)	9 (21.9)	5 (12.3)

**Table 2:** Baseline characteristics of patients with incidence of seizure and adverse effect.

Baseline characteristics	Over all (n = 75)	Levetiracetam (n = 28)	Lamotrigine (n = 2)	Valproate (n = 9)	Topiramate (n = 14)	Carbamazepine (n = 8)	Oxcarbazepine (n = 4)	Phenytoin (n = 2)	Phenobarbital (n = 6)	Rufinamide (n = 2)
Age										
1–12 months	6 (8)	4 (14.2)	0	0	1 (71)	0	0	0	1 (16.6)	0
1–7 years	39 (52)	16 (57.1)	1 (50)	5 (55.5)	6 (42.8)	3 (37.5)	3 (75)	2 (100)	3 (50)	0
8–14 years	30 (40)	8 ()28.5	1 (50)	4 (44.4)	7 (50)	5 (62.5)	1 (25)	0	2 (33.3)	2 (100)
Gender										
Female	32 (42.6)	10 (35.7)	1 (50)	4 (44.4)	7 (50)	4 (50)	1 (25)	1 (50)	3 (50)	1 (50)
Male	43 (57.3)	19 (67.8)	1 (50)	5 (55.5)	7 (50)	4 (50)	3 (75)	1 (50)	3 (50)	0
Epilepsy diagnosis										
Partial seizure	22 (29.3)	8 (28.5)	1 (50)	2 (22.2)	5 (35.2)	3 (37.5)	1 (25)	0	1 (16.6)	1 (50)
Generalized seizure	23 (30.6)	10 (35.7)	1 (50)	2 (22.2)	2 (14.2)	3 (37.5)	1 (25)	1 (50)	3 (50)	0
Other seizure type	8 (10.6)	3 (10.7)	0	1 (11.1)	2 (14.2)	1 (12.5)	0	1 (50)	0	0
Unclassified	22 (29.3)	7 (25)	0	4 (44.4)	5 (35.7)	1 (12.5)	2 (50)	0	2 (33.6)	1 (50)
Treatment history										
Newly diagnosed	11 (14.6)	7 (25)	0	1 (11.1)	0	1 (12.5)	1 (25)	1 (50)	1 (16.6)	0
Increasing the dose	27 (14.6)	11 (39.3)	1 (50)	4 (41.4)	7 (50)	2 (25)	0	0	3 (50)	0
Adding a modification	8 (36)	3 (10.7)	0	0	2 (14.2)	1 (12.5)	1 (25)	0	3 (50)	0
Maintenance dose	29 (10.6)	7 (25)	1 (50)	4 (44.4)	5 (35.7)	4 (50)	2 (50)	1 (50)	3 (50)	2 (100)
Treatment guidelines										
Followed BNF	40 (38.6)	7 (25)	2 (100)	6 (66.6)	12 (85.7)	7 (87.5)	2 (50)	0	3 (50)	1 (50)
Followed the practice	35 (33.3)	21 (75)	0	3 (33.3)	2 (14.2)	1 (12.5)	2 (50)	2 (100)	3 (50)	1 (50)

The physician followed the regular practice guideline (Table 2) in which 31 participants were selected during a 3 months’ period with a number (n = 35, 64.5%) of individualized AED prescribed. Out of 31 patients, 15 children were on monotherapy of levetiracetam. Patients on levetiracetam monotherapy were experiencing poor seizure control with a recorded number of seizure incidence (n = 10). The patient using a combination therapy of phenytoin and levetiracetam were experiencing some behavioral disturbance and sedation effect due to the synergistic side effect of this combination.

**Figure 2:**
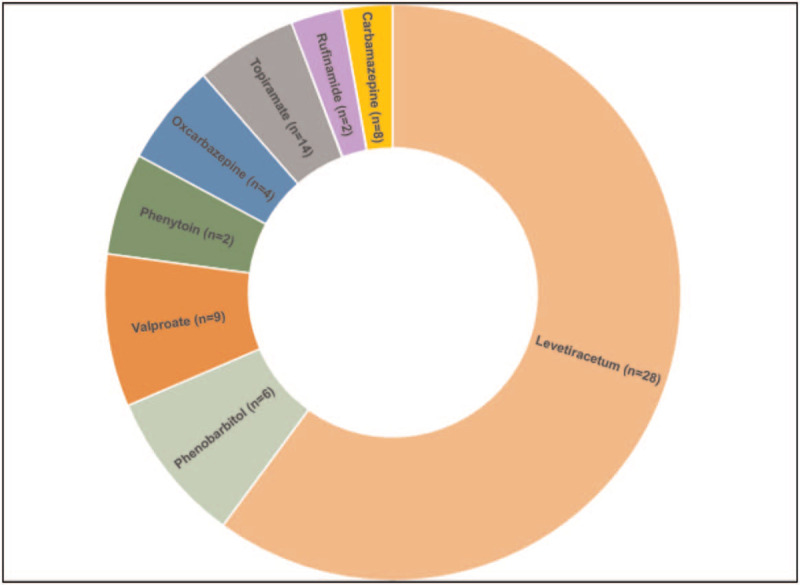
Patients treatment with less incidence of dizziness upon levetiracetam treatment.

**Figure 3:**
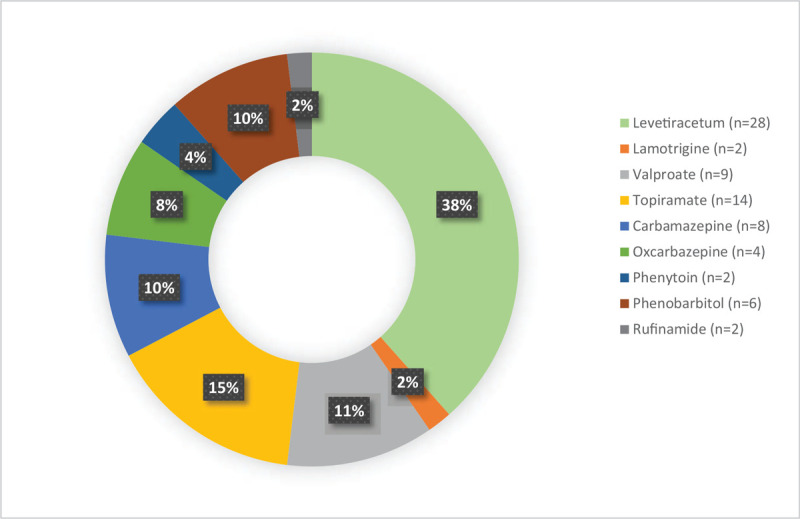
Patients treatment with less incidence of somnolence upon levetiracetam treatment.

The physician followed the BNF guideline and 17 participants were collected with (n = 40) number of individualized AED. A total incidence of seizure in patients who followed the BNF was significantly lower (n = 4) compared to the patients followed the regular practice (n = 17) a number of seizure incidence (Table 2). The most prescribed medication in this group were topiramate (n = 12), levetiracetam (n = 7), carbamazepine (n = 7), and valproic acid (n = 6). Patients who were in levetiracetam and who followed the BNF guideline recorded number of seizure incidence (n = 3). After 6 months of follow up we found that patient on levetiracetam were experiencing less adverse effect (n = 2) with no behavioral changes compared to the regular practice (n = 10). The side effects caused by the usage of antileptic drugs were shown in the Table 3. The most common side effects found were dizziness and somnolence. Patients that were on valproate had symptoms of nausea, dizziness and somnolence while patients who topiramate had weight loss, lethargy and anorexia. Comparing to the physician used a random guideline based on his/her preference, patients were experiencing less side effect while maintaining seizure control (Figs. 2 and 3).

**Table 3:** Dose information and its side effects of antiepileptic drugs in children.

Drugs	Initial dose (mg/kg/day)	Maintenance (mg/kg/day)	Daily dose (nos)	Side effects
Levetiracetam	10	20–60	2	Headache, anorexia, somnolence, behavioural problems
Lamotrigine	0.5	2.0–10.0	2	skin rash, somnolence, dizziness, nausea
Valproate	0.2	1.0–5.0	2	Nausea, dizziness, somnolence
Topiramate	1	6.0–9.0	2	Wt. loss, lethargy, anorexia
Carbamazepine	1	5.0–10.0	2	Dizziness, loss of appetite, ataxia, somnolence
Oxcarbazepine	5.0–8.0	10.0–30.0	2	Dizziness, ataxia, somnolence
Phenytoin	1.5	5.0–10.0	2	Cardiovascular risk, dermatoxic reaction, hepatic injury
Phenobarbital	15	5.0–10.0	2	Dizziness, ataxia, somnolence, headache, aggression
Rufinamide	10	1.0–5.0	1	CNS reaction, hypertension, multiorgan sensitivity, leukopenia

## 4 Discussion

Focusing on different antiepileptic studies that specifically examine the relationship between pediatric population and AEDs helped us to get a different perspective on managing an effective therapeutic control of the disease along with safety and tolerability. Because common AEDs usually produce adverse effects, titration of an AED often lessen these adverse effects and may quickly attain an effective or balanced dose for patients.^[17]^ Titration differs for each AEDs and may depend on several factors such as the process of therapy (monotherapy or adjunctive therapy), pharmacokinetic profile or the movement of the AED into, through and out of the body, the patient's response to treatment, adverse effect of AED, and potential drug-drug interactions.^[17]^

The present findings show that majority of the children were on monotherapy experience poor seizure control compared to patients who followed or in the BNF recommended guidelines. This study indicates that patients who were started on a small and slow dose titration as recommended by BNF guideline experience less adverse events while controlling their seizures. Previous study regarding efficacy, safety, and tolerability of AEDs stated that to improve safety and tolerability of some AEDS, requires slow and gradual dose titration.^[21]^ Starting the AED regimen at lower doses and titrating the dose for weeks is often appropriate to minimize the potential side effect like: sedation, dizziness, blurred vision, attention problems, headache or incoordination fatigue.^[22]^ However, more rapid titration is suitable for patient who is high risk and suffers from recurrent seizures.^[23]^

This study presents the patient using a combination therapy of phenytoin and levetiracetam experienced some behavioral disturbance and sedation effect. Previous studies found that a greater risk of drug toxicity is associated with combination therapy or polytherapy in pediatric patients particularly those receiving AEDs.^[24–26]^ In a prospective study of adverse drug reaction to AEDs in children found that more children who were in polytherapy developed up to a threefold of adverse drug reaction compared to children in monotherapy.^[25]^ Because majority of the AEDs share similar pharmacokinetic pathways, drug combination therapy may cause a major problem.^[27]^ For example, lamotrigine and valproic acid, in which lamotrigine glucuronidation inhibits by valproic acid resulting to increase plasma concentration and toxicity.^[25]^ This finding is in line with the results of a review study in which the combination of lamotrigine and valproate (valproic acid) increased a possibility of side effects.^[27]^ Although, combination of AEDs was hindered by side effects, the idea of polytherapy is to improve seizure control and tolerability of the treatment and to obtain better control of the refractory seizure.^[26,27]^ In addition, newer AEDS are often associated with safety and efficacy resulting to the requirement of expanding therapeutic options.^[17,28]^

The dissimilarities among the two groups, that the group who followed the regular practice were more likely to suffer from side effects of the medication due to initiating the medication with a maintenance dose or high dose without titration period or initiating medication is not suited for the patient age or epilepsy syndrome. On the other hand, the group of patients who followed the BNF guideline experienced minimal adverse effect with seizure control and little dose adjustment needed in the population. We have learned from history, and through the application of dose titration, that the use of AEDs implies significant variability specially in pediatric population, regarding dosing and adverse effect due to several changes in pharmacokinetic and pharmacodynamic responses occur with maturation throughout childhood. As a result of this study, a lack of guidance regarding initiation and dose titration of AED monotherapies or a novel adjunctive AED's into polytherapy regimen is still a major concern in pediatric population and a large scale, multicenter efforts are needed to design, conduct a future clinically relevant randomized clinical trials that address the efficacy and tolerability of AEDs dose titration.

The findings of this study present some limitations. First, the findings cannot be generalized because it is conducted in a single-center study. Other limitations such as physiological aspects like circadian rhythms and metabolic changes or disorders were not recorded in the medical records. The study therefore cannot strongly ascertain the effect of antiepileptic drugs among pediatric children with epilepsy in Saudi Arabia. However, these findings may add value to the limited literature and form preliminary data of the effect of antiepileptic drugs among pediatric children with epilepsy in Saudi Arabia. This study nevertheless is one of the most recent prospective cohort studies in Saudi Arabia focusing the effects of antiepileptic drugs.

In conclusion, the group who followed the regular practice found having a greater incidence of documented adverse effect compared to the patients following the BNF guideline. The titrating antiepileptic medication have a detrimental effect on the pediatric population as observed in this study. Further study to design local guideline in PSMMC is essential to develop the rational use of antiepileptic drugs in children.

## Acknowledgments

The authors extend their appreciation to the College of Medicine Research Centre, Deanship of Scientific Research, King Saud University, Riyadh, Saudi Arabia for funding this work.

## Author contributions

**Conceptualization:** Badriyah S. Alotaibi, Brahim Tabarki Melaiki, Aljawharah Mohammad Almadhi, Lulwah Haitham Alfares, Nahlah Ahmed Alalkami, Abdulaziz Alodhayani.

**Data curation:** Abdulaziz Alodhayani, Nahlah Ahmed Alalkami.

**Formal analysis:** Badriyah S. Alotaibi.

**Funding acquisition:** Nahlah Ahmed Alalkami.

**Investigation:** Ashraf Alwan, Khalid Nijr Alotaibi, Brahim Tabarki Melaiki, Aljawharah Mohammad Almadhi, Nahlah Ahmed Alalkami.

**Project administration:** Khalid Nijr Alotaibi.

**Supervision:** Abdulaziz Alodhayani, Badriyah S. Alotaibi.

**Writing – original draft:** Badriyah S. Alotaibi, Ashraf Alwan, Khalid Nijr Alotaibi, Brahim Tabarki Melaiki, Aljawharah Mohammad Almadhi, Lulwah Haitham Alfares.

**Writing – review & editing:** Abdulaziz Alodhayani.

## Supplementary Material

Supplemental Digital Content
