# Supplementary material for: Detrimental effect of antiepileptic drugs dose in pediatric children with epilepsy in Saudi Arabia: A prospective cohort study
Source: Medicine (Baltimore). 2021 Jul 2;100(26):e26478. doi: 10.1097/MD.0000000000026478 (PMC8257875; doi:10.1097/MD.0000000000026478)
Supplement: Supplemental Digital Content [file medi-100-e26478-s001.pdf]

Prince Sultan Military Medical City
Research Center

Patient information :

Date :

Medical ID #

Weight (kg) :

Age:

Gender:

Diagnose :

Seizures type :

Number of attacks :

Current medication:

Medication	Dose (mg/kg) frequency	Starting dose	Date of starting the medication	Comment	Per guideline
